# Symblepharon as Ocular Manifestation Post Stevens-Johnson Syndrome: A Rare Case

**DOI:** 10.22336/rjo.2024.84

**Published:** 2024

**Authors:** Edy Wibowo, Regina Vika Maharani, Nadya Adiwijaya Sutikno

**Affiliations:** 1 Opthalmology Department, Bethesda Hospital Yogyakarta, Indonesia; 2 Universitas Kristen Duta Wacana, Faculty of Medicine, Yogyakarta, Indonesia

**Keywords:** ocular, symblepharon, Stevens-Johnson syndrome, MMP = Mucous Membrane Pemphigoid, OCP = Ocular Cicatricial Pemphigoid, SJS = Stevens-Johnson syndrome

## Abstract

**Background:**

Stevens-Johnson syndrome (SJS) is a life-threatening condition resulting from a severe reaction to the use of certain drugs, with the highest incidence found in children. It manifests as a triad of skin, orifice, and ocular mucosa lesions. Ocular manifestations most commonly involve the conjunctiva and eyelids. This case report further discusses symblepharon as an ocular manifestation of SJS.

**Method:**

A case report.

**Case report:**

A 10-year-old boy came with decreased vision and an inability to produce tears. On examination, pseudomembranous conjunctivitis was found in both eyes, granulation tissue in the right eye, and erosion of the corneal epithelium in the left eye. The posterior segment could not be assessed due to symblepharon. It was known that the patient previously experienced SJS in early 2023. Symblepharectomy was carried out with the indication of separate adhesions caused by symblepharon.

**Discussion:**

Symblepharon is a rare, severe ocular manifestation of SJS (Stevens-Johnson syndrome). Previous studies found that severe ocular occurred in around 4% and 11.1% of cases. This happened because of ongoing chronic inflammation due to SJS. Symblepharon is an adhesion of eyelids and bulbar conjunctiva, which can harm the eye because it can cause cicatricial then disruption of the tear film meniscus, limit eye mobility, and cause visual disturbances.

**Conclusion:**

Symblepharon occurs due to prolonged inflammation, which can structurally and functionally disrupt the eye. Early discovery of symblepharon, especially in severe manifestations of SJS, can help prevent further damage to the eye.

## Introduction

Stevens-Johnson syndrome (SJS) is a life-threatening mucocutaneous disorder resulting from a severe reaction to the use of drugs such as antibiotics (amoxicillin, sulfonamide group), antiepileptics (phenobarbital, phenytoin), or anesthetic drugs (fentanyl) [1]. The incidence of SJS in the general population each year from 1995 to 2013 was up to 5.76 cases per one million people in the world and mainly occurred in patients aged 1-10 years or 80 years and over [2]. SJS can cause reactions throughout the body, including the eye membrane. According to the previous study, ocular manifestations due to SJS mostly involved the conjunctiva (81.4%) and eyelids (74%) [2]. Inflammatory manifestations in SJS can continue until they become chronic, such as symblepharon [1]. Symblepharon was classically described for the first time by Fuchs (1892) as a cicatricial adhesion between the eyelid and the conjunctiva. Symblepharon is a complex problem in ocular cases. It can cause limited eye movement, an inability to blink, entropion, ptosis, and secondary ocular surface damage that can affect the cornea and disrupt vision. The etiology can occur exogenously, such as thermal or chemical burns and pterygium, or endogenously, for example, due to Stevens-Johnson syndrome (SJS), ocular cicatricial pemphigoid (OCP) or mucous membrane pemphigoid (MMP), and keratoconjunctivitis sicca or dry eyes [3].

## Case report

**Fig. 1:**
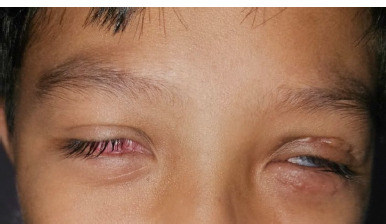
Both eyes are narrowed and cannot open optimally. Cicatrices and granulation tissue appear to cover the anterior segment of the right eye, and narrowing and post-palpebral graft scars on the left eye

**Fig. 2:**
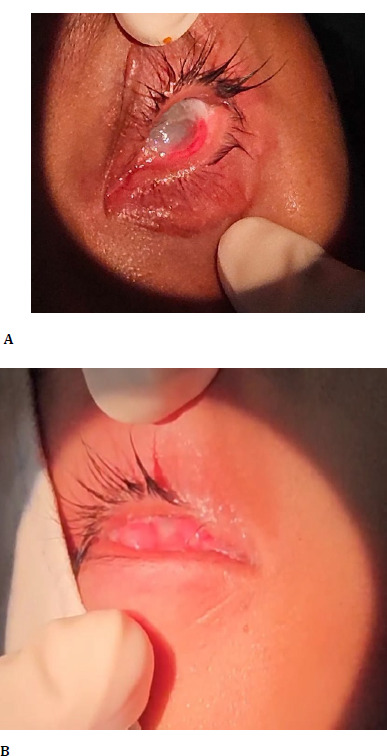
**A**. The left eye has grade IIIc3+ symblepharon with corneal epithelial erosion. **B**. The right eye has grade IVc3+ symblepharon with granulation tissue and edema originating from the eyelid

A 10-year-old boy came with a blurry left eye. The patient complained of narrowing the right and left eyes and decreased vision in the left eye. He also complained of not being able to produce tears. The right eye’s vision could not be assessed because it was obscured by granulation tissue and symblepharon, and the left-right vision was 1/300. The left eye field of view was narrow. Symblepharon grade IVc+ was observed in the right eye and grade IIIc+ in the eyes. There were no complaints of pain. Both anterior segments of the eyes appeared reddish and slightly edematous. Palpebral tissue appeared to form granulations in the anterior segment of the right eye. Pseudomembrane conjunctivitis was also found in both eyes. Corneal epithelial erosion was found in the left eye. The posterior segment of the eye could not be examined due to the adhesion of the upper and lower palpebral conjunctiva to the bulbar conjunctiva. Vital signs and blood tests were within normal limits. An allergy examination showed no allergies to fentanyl, rocuronium, ketorolac, ketamine, atracurium, propofol, amoxicillin, glyceryl guaiacolate, chlorpheniramine maleate, bromhexine, paracetamol, acetylsalicylic acid, or ibuprofen. The patient had a history of SJS in March 2023 with the manifestation of a severe rash on the skin throughout the body, especially the face, chest, and extremities, and then ocular manifestations appeared in the form of symblepharon. Until now, no definite cause has been found for SJS in patients. Because the drops were located far from a tertiary, the only eye treatment given was antibiotic drops, and the complaints became worse, so the patient was then referred to a higher-level hospital. The patient underwent eyelid and conjunctival repair of the right eye in October 2023. The patient was then diagnosed with five symblepharon post-SJS. As initial treatment, the patient was given sodium diclofenac 0.5 mg, ibuprofen 400 mg, cetirizine 5 mg, Cendo Hyalub (sodium hyaluronate) four times a day, one drop, Cendo Lyteers (sodium chloride and potassium chloride) three times a day, one drop, and moxifloxacin 0.5% six times a day, one drop. The patient underwent a symblepharectomy to separate the adhesions, which was carried out by administering local anesthesia (pantocaine 0.5%) and then rinsed with Miriwash. The adhesions were separated slowly to improve mobilization of the conjunctival area and open the lacrimal duct so that tears could come out. After the procedure, there was an improvement in the patient’s vision, the right eye’s vision becoming 1/300, the left eye’s vision becoming 1/300, and the visual field of both eyes also becoming wider. The patient’s family was trained to maintain the hygiene of the patient’s eyes. The patient was scheduled for follow-up every 2 days for irrigation after returning home from hospitalization (**Fig. 1**). Both eyes were narrowed and could not open optimally. It appeared that the anterior segment of the right eye was covered by cicatrices, granulation tissue, and narrowing and post-palpebral graft scars on the left eye (**Fig. 2 A, B**). The left eye had grade IIIc3+ symblepharon with corneal epithelial erosion, and the right eye had grade IVc3+ symblepharon with granulation tissue and edema originating from the eyelid.

## Discussion

Symblepharon is a severe ocular manifestation that can be experienced in SJS. Previous studies stated that severe ocular involvement occurred in around 11.1% of cases [2] and 4% [4], where symblepharon is rare in SJS cases. Based on the clinical level, ocular manifestations in SJS are divided into acute, subacute, and chronic. The acute stage usually occurs within 2 weeks after onset and consists of conjunctivitis, conjunctival hemorrhage, pseudomembrane formation in the conjunctiva, meibomianitis, symblepharon, and defects in the ocular epithelium. In the acute phase, keratosis is apoptotic and causes inflammatory effects and the loss of epithelium on the eye’s surface. Eyelid edema and conjunctival injection are ocular manifestations in the acute phase. Even though the skin lesions have healed, ongoing inflammation causes chronic cicatricial conjunctiva, trichiasis, and uneven eyelid margins to become persistent manifestations, spreading to other parts, such as the meibomian glands and lacrimal glands. Continuous inflammation will cause chronic manifestations in the eye, which in the end can cause visual disturbances such as symblepharon, permanent ankyloblepharon, cicatricial entropion, and keratinization of the edges of the eyelids, tarsals, and the surface of the bulbar conjunctiva. These manifestations cause disruption of the tear film meniscus, inhibit proper eyelid closure and blinking, and sometimes limit eye mobility [2,5]. In this case, the patient experienced chronic symblepharon, which occurred from March 2023 until surgery was carried out in October 2023 and recurred again in July 2024. Symblepharon formation results from adhesions of the eyelids and bulbar conjunctiva. Early prevention that can be done is to use glass rods to prevent the formation of symblepharon. Still, these devices do not separate the surfaces when the epithelium begins to heal, so conjunctival tears may cause ongoing inflammation. A doctor can also use a silicone lens placed in the fornix for 2 weeks to prevent symblepharon formation. However, it is less effective in symblepharon, which occurs due to chemicals where fibrosis of the conjunctiva and scar tissue can cause the fornix to be shortened. A silicone lens can provide temporary comfort, although adhesions can recur after removing the ring. Scarring symblepharon can be repaired for the palpebral membrane and conjunctiva with a graft from the inner lip mucosa placed on the removed scar. Unfortunately, in recent studies, there has been no truly effective treatment for progressive conjunctival cicatrices, where recurrence of adhesions often occurs [3,6]. As many as 30-50% of patients who experience SJS at the time of visit show chronic ocular abnormalities. Early follow-up should be carried out within the first month after discharge from hospitalization and repeated every 2-4 months in the first year and then at least 6 months after that to evaluate the eye condition. Acute treatment of chronic complications, such as in the presented case, aims to prevent and treat other disorders, such as dry eye disease, palpebral malposition, irregular eyelashes, keratinization of the posterior lid edges, conjunctival abnormalities, and corneal abnormalities [7].

## Conclusion

We reported a rare case of symblepharon post-SJS. Symblepharon occurs due to prolonged inflammation, disrupting the eye’s anatomy and function. Early detection can help prevent ocular manifestations from becoming severe.
